# Supplementary material for: Survey on Sheep Usage in Biomedical Research
Source: Animals (Basel). 2020 Aug 30;10(9):1528. doi: 10.3390/ani10091528 (PMC7552153; doi:10.3390/ani10091528)
Supplement: Supplementary file 1 [file animals-10-01528-s001.pdf]

Sheep for Biomedical Research

About 30 000 sheep are enrolled in research projects each year in EU member countries all together. In contrast to mice, rats and rabbits, sheep are not specifically bred for research. Our aim is to evaluate the sheep selection criteria(s) and to see, if there is a need to change/ improve the current situation in order to refine studies using sheep.

Thank you in advance for answering the following questions!

1. For how long have you worked with sheep as research animals?

- ◯ <1 year
- ◯ 1-5 years
- ◯ >5 years

2. What is your role at your institution/company? (multiple answers possible)

- ☐ veterinarian
- ☐ animal care giver/technician
- ☐ scientist/researcher
- ☐ manager

other (please specify)

____

3. For how long has your institution/company worked with sheep?

- ◯ <1 year
- ◯ 1-5 years
- ◯ >5 years

4. How many sheep do you use/year?

- ◯ 1-20
- ◯ 21-50
- ◯ 51-100
- ◯ >101

5. What is your main field of research with sheep? (multiple answers possible)

- [ ] behavior
- [ ] physiology
- [ ] surgery
- [ ] testing medical devices

other (please specify)

6. Where do you purchase your sheep from?

- ◯ a (local) farm
- ◯ a commercial breeder
- ◯ own flock

7. Which gender are your sheep? (multiple answers possible)

- [ ] intact males
- [ ] neutered males
- [ ] females
- [ ] not important/depending on availability

8. What is the preferred age range of the sheep at the beginning of your study? (multiple answers possible)

- [ ] <1 year old
- [ ] 1 - 2 years old
- [ ] >2 years old
- [ ] not important/depending on availability

9. What are the main criteria for choosing a sheep supplier (5: very important, 1: not important)?

	5	4	3	2	1
availability of the animals	☐	☐	☐	☐	☐
distance from your facility	☐	☐	☐	☐	☐
health status	☐	☐	☐	☐	☐
animal homogeneity/uniformity	☐	☐	☐	☐	☐
provider (trust, experience)	☐	☐	☐	☐	☐
price	☐	☐	☐	☐	☐

other important criteria (please specify):

10. Do you prefer a certain breed for your research?

- ○ no
- ○ yes (please specify breed and reason)

11. Do you have a health monitoring program for your sheep (e.g. according to FELASA)?

- ○ I don't know
- ○ no
- ○ yes (please specify what you are monitoring)

12. Do you vaccinate your sheep?

- ○ I don't know
- ○ no
- ○ yes (please specify against what)

13. Have you ever encountered problems in your sheep, not related to the experimental protocol (e.g. health issues)?

- I don't know
- no
- yes (please specify which ones)

14. How much do you pay for your sheep from your current provider?

- I don't know
- please specify price and currency

15. How much are you willing to pay for a sheep with a controlled health status from a commercial breeder?

- I don't know
- please specify maximum price and currency

16. Do you work at:

- an academic institution?
- a private company?
- other? (please specify)

17. Would you like to be contacted by the authors of this questionnaire?

- no
- yes (please enter your e-mail address that will be kept confidential)

18. Comments
